# Evaluation of oxidative stress in endometriosis: A case-control study

**Published:** 2015

**Authors:** Maryam Alizadeh, Soleiman Mahjoub, Seddigheh Esmaelzadeh, Karimollah Hajian, Zahra Basirat, Maryam Ghasemi

**Affiliations:** 1 Department of Biochemistry and Biophysics, Faculty of Medicine, Babol University of Medical Sciences, Babol, Iran.; 2 Fatemeh Zahra Infertility and Reproductive Health Research Center, Babol University of Medical Sciences, Babol, Iran.; 3 Department of Epidemiology, Babol University of Medical Sciences, Babol, Iran.

**Keywords:** Endometriosis, Oxidative stress, Iron, Malondialdehyde, Carbonyl.

## Abstract

**Background:**

Recent studies have suggested that oxidative stress (OS) may have a contribution in the pathogenesis of endometriosis. However, the results of previous studies regarding OS in endometriosis are controversial. The objective of this study was to compare the serum levels of OS markers in endometriosis versus the control group.

**Methods:**

This case-control study was carried out on 30 women with endometriosis aged 20-38 years presented to Fatemeh Zahra Infertility and Reproductive Health Research Center, Babol, Iran between March 2011 through November 2013. The serum samples of 40 women with same age were collected as the control group. The serum levels of malondialdehyde (MDA), carbonyl and iron were measured by photometric methods and compared between the patient and control groups using t-test. Also, we used ROC curve analysis to determine the discrimination ability of these markers.

**Results:**

Serum iron in endometriosis patients was significantly higher than control (p<0.0001). Area under the ROC curve (AUC) for iron, MDA and carbonyl were 0.899, 0.648 and 0.530, respectively. Serum iron at cutoff value of 173.3 µg/dl exhibited high discrimination ability to discriminate endometriosis from control.

**Conclusion:**

These findings indicate that the high level of serum iron may promote OS in patients with endometriosis. In addition, serum Iron at cut off level of 173.3 exhibits high discriminative ability to distinguish patients with endometriosis from healthy subjects.

Endometriosis is a common benign gynecologic disease which is usually associated with pelvic pain and even infertility in women (1). This condition is defined as the presence of ectopic endometrial tissue outside the uterine cavity (2). The prevalence rate of this disease is 5-15% in women of reproductive age (3). Nowadays, endometriosis is known as one of the leading causes of infertility in women and the incidence of this disease is 35 to 50% (4). The etiology of endometriosis is not clear but some general risk factors such as smoking, alcohol use and low body mass index may have a role in the development of this condition (5). Recent studies have shown that (OS) and reactive oxygen species (ROS) may have had a contribution (6). ROS may alter the properties of endothelial cells such as permeability and adhesion molecule expression and as a result, leads to the dissemination of the inflammatory process (3). OS substances may have a contributive role in the pathogenesis of endometriosis through the activation of macrophages. These activated macrophages can aggravate oxidative stress conditions by the production of lipid peroxides and other by-products from reaction between apolipoproteins and peroxides.

The sum of these events increased the concentrations of pro-inflammatory mediators and induce inflammatory conditions of women with endometriosis (7). Oxidative stress is the excess production of free radicals and ROS compared to the neutralization of these compositions by antioxidants (8). One of the potential pro-oxidant is iron. Free or catalytic form of iron produces ROS by Fenton reaction and there inducing oxidative stress (9). Iron is released from metabolism of hemoglobin and heme by macrophages. Several studies have indicated high level of iron in peritoneal cavity of women with endometriosis and concentration of iron in peritoneal fluid is dependent to severity of the disease (10, 11). 

Malondialdehyde (MDA) is a common indicator for estimating the level of lipid peroxidation in biological samples, measured by reaction with thiobarbituric acid to produce red complex (12). Protein carbonyl group is a biological marker of oxidative stress (13, 14). The objective of the present study was to evaluate the levels of main oxidative stress markers such as MDA, protein carbonyl and iron in serum of women with endometriosis to compare with control group.

## Methods

**Study Population:** A case-control study was carried out on 30 women with endometriosis who referred to Fatemeh Zahra Infertility and Reproductive Health Research Center, Babol, Iran from March 2011 to November 2013. Also, 40 matched healthy women served as the control group. Exclusion criteria included the presence of infectious diseases such as hepatitis or anemia. The study protocol was approved by the Ethics Committee of Babol University of Medical Sciences and informed consent was obtained. The primary objective of this study was to compare the serum levels of the OS markers between patients and control. The secondary objective was to determine the discriminative ability of each marker for the differentiation of patients from control. Blood samples were collected in sterile tubes and centrifuged at 3000 rpm for 20 mins. Then, the collected sera were stored at -80^o^C until assessment. 

**Laboratory methods:** In this study, we measured three markers of oxidative stress in the serum of 30 women with endometriosis and 40 women as the control group. The markers were MDA, carbonyl and iron. Before the experiments, the samples were defreezed in 4^o^C.

**TBARS assay:** Malondialdehyde (MDA) concentration in serum case and control were measured by TBARS (thiobarbituric acid reactive-substances) assay. Basing on this assay is a reaction between MDA and thiobarbituric acid. The absorbance of samples was recorded against blank at wavelength of 535 nm (15). Concentration of MDA was reported in nmol/ml. The rate reaction is depending to temperature and thiobarbituric acid concentration. Thiobarbituric acid also reacts with conjugated aldehydes and hydroperoxides (16).

**Carbonyl assay:** The levels of carbonyl were determined based on reaction between 2, 4-dinitrophenylhidrazine and carbonyl by Levine et al. method (17). This reaction causes to produce 2, 4-dinitrophenylhidrazone that can be determined by spectrophotometer in 370 nm. Due to reduced amount of protein during washing steps, we calculated it in the final step. Sample proteins were measured by a commercial kit of total protein (ZIESTCHEM.CO, Iran, Tehran). Carbonyl content was expressed as nmol/mg protein (14).

**Measurement of serum Iron:** The concentrations of iron in the serum of 30 patients and 40 controls were determined by ferrozine method using commercial kit (Darman Kave Co., Iran). In this method, iron, which is bounded proteins in buffer (PH 4.5) was released to free form while the proteins are not precipitated. Free iron is reduced and produced purple-colored complex with ferrozine. Intensity of this color is related to the concentration of serum iron and determined by a spectrophotometer (Jenway UV/VIS, 6505 model, UK).

**Statistical Analysis:** Data were analyzed by the Statistical Package for Social Sciences (version 18; SPSS). Results were presented as the mean and standard error for each marker in the serum of case and control. The data were analyzed using t-test and Pearson correlation test. A p-value <0.05 was considered statistically significant. The receiver operative characteristic (ROC) curve was drawn, and the area under that curve (AUC) was calculated, as 95% CI to detect the best cut-off value for the index. The optimal cut-off value that presented the highest sum of sensitivity and specificity was established. 

## Results

The mean value of serum iron in the patients was higher than control. There was a significant difference (p<0.0001) in the serum iron of patients compared with control (table 1). 

**Table 1:** Mean±S.E of oxidative stress markers in serum of endometriosis patients and control group.

Markers	Mean±S.E	P Value
MDA(nmol/ml)	3.31±0.07 3.23±0.09	P>0.05
Case(n=30) Control(n=40)		
Carbonyl (nmol/mg _pro_)	0.84±0.19 0.54±0.08	P>0.05
Case(n=30) Control(n=40)		
Iron (µg/dl)	172.24±17.45 86.76±5.91	P<0.0001

**Figure 1:**
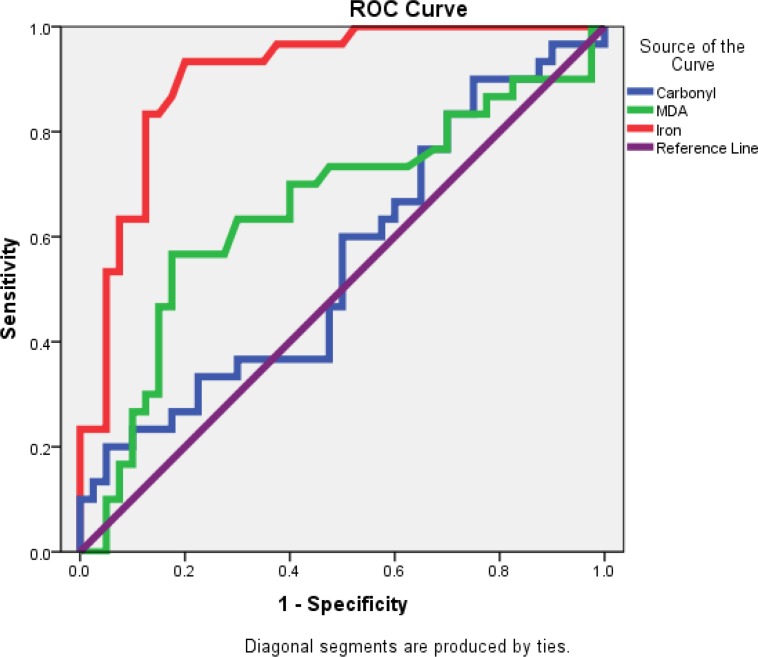
ROC curve of oxidative stress markers for serum of patients with endometriosis vs control group

The areas under ROC curve (AUC) in serum of endometriosis for iron, MDA and carbonyl were 0.899, 0.648 and 0.530, respectively (figure 1 and table 2). In ROC curve analysis, only serum iron with an AUC value of 0.899 demonstrated highly significant discriminative ability at cut-off value of 173.3 µg/dl.

**Table 2:** Area under the ROC curve (AUC) of oxidative stress markers in serum of endometriosis patients and control group.

Variables	AUC	Asymptotic Sig.	Asymptotic 95% CI	
			Lower Bound	Upper Bound
MDA	0.648	0.071*	0.509	0.787
Carbonyl	0.530	0.073	0.387	0.672
Iron	0.899	0.039**	0.822	0.975
				

* P< 0.05,

** P< 0.0001

## Discussion

The results of this study indicate that the level of serum iron as pro-oxidant marker in patients with endometriosis was significantly higher than the control group. Increase of serum MDA and carbonyl in the patients with endometriosis were not significant. Recent studies have demonstrated the presence of iron overload in various components of the peritoneal cavity of endometriosis patients such as peritoneal fluid, macrophages, and endometriotic lesions; which strongly suggests disruption of iron homeostasis in the peritoneal cavity of patients (18, 19). 

In endometriosis patients, iron overload may originate from lysis of pelvic erythrocytes (20). Retrograde menstruation is considered an essential step in the pathogenesis of peritoneal endometriosis, according to Sampson’s theory. This reflux, transporting menstrual endometrial tissue through the fallopian tubes into the peritoneal cavity, is a common physiologic event in all menstruating women with patent tubes (21). Severe hemolysis during retrograde menstruation, along with a defective or overwhelmed peritoneal disposal system in the case of increased menstrual reflux, results in iron overload in the peritoneal environment, which in turn permits attachment and growth of the endometrial cells or fragments. This iron accumulation may have numerous cytotoxic effects as it disrupts the balance between free radicals production and antioxidant defense, which leads to oxidative stress (OS) implicated in the pathogenesis of endometriosis (22).

Iron toxicity is mainly related to its ability to catalyze the production of a wide variety of free radical damaging species, leading to the deregulation of cellular processes, cell dysfunction, and eventually to apoptosis or necrosis through lipid peroxidation, protein, and DNA damage (23, 24).

Most studies considered iron overload in peritoneal fluid of endometriosis patients, but we investigated the level of iron in serum of the patients and matched healthy subjects. Our finding confirms iron overload in patients with endometriosis. 

Iron overload in the peritoneal fluid provokes oxidative injury and inflammatory response, involving peritoneal macrophages in particular, which promotes the proliferative capacity of ectopic implants of endometrium in the peritoneal cavity (20, 25). Oxidative injury occurs when continued delivery of iron to the peritoneal macrophages is associated with inhibition of iron storage in ferritin. In normal condition, specific proteins such as ferritin and transferrin iron stores, prevent unfavorable reactions between iron and oxygen radicals, but this balance is disturbed in pathological condition (11).

In our study, the average concentration of MDA and carbonyl was higher in the serum of the patients compared to control, but no significant differences were observed for these markers between two groups. Earlier studies showed that lipid peroxide concentration was highest in peritoneal fluid samples of infertile patients with endometriosis compared with women with idiopathic infertility (25). 

According to the findings of this study, the concentration of iron in the serum of patients was significantly higher than the control group. In normal status, specific proteins such as transferrin iron stores prevent unfavorable reactions between iron and oxygen radicals, but this balance is disturbed in pathological status (11). Overload of iron in peritoneal cavity of patients with endometrioma may be attributed to activity of macrophages in pelvic cavity and destruction of heme and hemoglobin (24). 

Some researchers recently have demonstrated increased iron storage in the peritoneal macrophages of endometriosis patients compared with healthy subjects, correlating with iron load in peritoneal fluid (18). The cause of high level of iron in patients with endometriosis compared with healthy subjects could be due to retrograde menstruation that carries highly pro-oxidant factors such as heme and iron (26). Previous studies showed that increase of hemoglobin destruction and producing free iron is a strong stimulator for increase oxidative stress (11). Indeed, iron can act as a catalyst to potentiate oxygen toxicity through the generation of a wide range of free radical species, including hydroxyl radical (OH^o^). Hydroxyl radicals are the most reactive free radical species known and have the ability to react with a wide range of cellular constituents, including amino-acid residues and purine and pyrimidine bases of DNA, as well as attacking membrane lipids to initiate a free radical chain reaction known as lipid peroxidation (18).

On the other hand, the increase of peroxidation metabolites and oxygen radicals were produced by this reaction also intensifying degradation of hemoglobin and as a result of iron concentration. Therefore, exacerbation of oxidative stress in this state is bidirectional and oxidative stress in time lapse was higher (11). In the present study, high area under ROC curve (AUC) for iron as an important pre-oxidant factor may be a good factor to distinguish the endometriosis patients from healthy subjects.

In conclusion, the results of the present study indicate a significant relationship between iron and the lipid peroxidation marker. High concentration of iron may promote OS in patients with endometrioma. Also, based on the ROC curve results, iron showed high discrimination ability to distinguish endometriosis patients from healthy subjects. 

## Notes

**Funding:** The authors are grateful to Babol University of Medical Sciences for their support.
